# The Role of High Flow Nasal Cannula in COVID-19 Associated Pneumomediastinum and Pneumothorax

**DOI:** 10.3390/healthcare9060620

**Published:** 2021-05-22

**Authors:** Francesca Simioli, Anna Annunziata, Giorgio Emanuele Polistina, Antonietta Coppola, Valentina Di Spirito, Giuseppe Fiorentino

**Affiliations:** Sub-intensive Care Unit, Department of Respiratory Pathophysiology and Rehabilitation Monaldi–A.O. Dei Colli, Via Gaetano Quagliariello 54, 80131 Naples, Italy; anna.annunziata@gmail.com (A.A.); giorgiopolistina@gmail.com (G.E.P.); antonietta.coppola84@gmail.com (A.C.); valentinadispirito@hotmail.com (V.D.S.); giuseppefiorentino1@gmail.com (G.F.)

**Keywords:** critical COVID-19, non-invasive ventilation, mechanical ventilation, ARDS, P-SILI

## Abstract

Background: Pneumomediastinum, subcutaneous emphysema and pneumothorax are not rarely observed during the COVID-19 pandemic. Such complications can worsen gas exchange and the overall prognosis in critical patients. The aim of this study is to investigate what predisposing factors are related to pneumomediastinum and pneumothorax in SARS-CoV2-Acute Respiratory Distress Syndrome (ARDS), what symptoms may predict a severe and potentially fatal complication and what therapeutical approach may provide a better outcome. Methods: In this single center cohort study, we recorded data from 45 critically ill COVID-19 patients who developed one or more complicating events among pneumomediastinum, subcutaneous emphysema and pneumothorax. All patients showed ARDS and underwent non-invasive ventilation (NIV) at baseline. Patients with mild to moderate ARDS and pneumomediastinum/pneumothorax (*n* = 25) received High Flow Nasal Cannula (HFNC), while patients with severe ARDS and pneumomediastinum/pneumothorax underwent HFNC (*n* = 10) or invasive mechanical ventilation (IMV) (*n* = 10). Results: Pneumomediastinum/pneumothorax developed in 10.5% of subjects affected by SARS-coV2-ARDS. Dyspnea affected 40% and cough affected 37% of subjects. High resolution computed tomography of the chest showed bilateral diffuse ground glass opacities (GGO) in 100% of subjects. Traction bronchiolectasis, reticulation, crazy paving and distortion were observed in 64%. Furthermore, 36% showed subcutaneous emphysema. Non-severe ARDS cases received HFNC, and 76% patients recovered from pneumomediastinum/pneumothorax over a median follow up of 5 days. Among severe ARDS cases the recovery rate of pneumomediastinum/pneumothorax was 70% with the HFNC approach, and 10% with IMV. Conclusion: HFNC is a safe and effective ventilatory approach for critical COVID-19 and has a positive role in associated complications such as pneumomediastinum and pneumothorax.

## 1. Background

COVID-19 is an emerging infectious disease caused by a novel coronavirus named SARS-CoV2. As the global pandemic progresses, novel presentations and uncommon complications are recognized [1]. Multiple case reports are available about spontaneous or iatrogenic pneumomediastinum and pneumothorax related to SARS-CoV2 [2,3,4]. Which factors are underlying these conditions remain unclear. Lastly, no data are available about what approach can ameliorate the outcome of these patients.

High Flow Nasal Cannula (HFNC) is a ventilatory support able to deliver a high flow of properly heated and humidified air. It provides a reliable oxygen dispatch and allows an elevated fraction of inhaled oxygen (FiO2), from 21% to virtually 100%, by preventing the dilution of oxygen with room air. The relatively small interface and the physiological temperature of this therapy is responsible for a very high tolerance. Overall compliance is greater than non-invasive ventilation (NIV). HFNC demonstrated to improve oxygenation, work of breathing, respiratory rate and dyspnea scores in patients with hypoxemic respiratory failure [5]. Augmented work of breathing can be estimated based on respiratory rate and impaired thoraco-abdominal synchrony. These are signals of an acute respiratory distress that may lead to invasive or non-invasive ventilation. Lee et al. reported how HFNC improves signs of a distressed breathing and improves oxygenation. The authors conclude that HFNC reduces the need for endotracheal intubation, thus no evidence supports an effect on mortality.

During this COVID-19 pandemic, HFNC was widely used to support respiratory failure in critically affected patients, though there are no clinical trials clarifying its safety and efficacy [6]. Some concerns arose about initiating HFNC in patients affected by acute respiratory failure since prolonged hypoxemia may lead to worse outcomes, and, besides, it may delay the escalation to ventilation [7]. The adoption of HFNC as a primary or rescue therapy for respiratory failure secondary to COVID-19 has led to similar concerns [8]. As hypoxemic normocapnic respiratory failure is widely accepted to be an indicator for HFNC use, its use has been extensive for COVID-19 since the first step of the current pandemic. Pneumomediastinum and pneumothorax are not currently indicators for HFNC use. Limited data are reported about the consequences of using HFNC in subjects with concomitant pneumothorax. Baudin et al. described 177 HFNC episodes involving 145 subjects. Among this population, six preexisting pneumothoraces (3%) were identified before HFNC initiation, none of which worsened under HFNC. Two episodes (1%) of new pneumothoraces occurred [9].

## 2. Aims and Scope

The aim of this study is to investigate what predisposing factors are related to pneumomediastinum and pneumothorax in SARS-CoV2-ARDS, what symptoms may predict a severe and potentially fatal complication and what therapeutical approach may provide a better outcome.

## 3. Methods

This is a single center cohort study about use of HFNC in complicated COVID-19. It included 45 patients selected among those admitted to our department from August 2020 to January 2021 for critical COVID-19. Inclusion criteria were: SARS-CoV-2 infection confirmed by reverse-transcriptase–polymerase-chain-reaction assay, interstitial pneumonia assessed by a high-resolution computed tomography (HRCT) of the chest and acute respiratory distress syndrome (ARDS). In addition, patients were required to develop a complicating event such as pneumomediastinum and/or pneumothorax. All patients provided informed consent. All patients received the pharmacological standard of care, and a ventilatory support. Continuous positive airway pressure (CPAP) or HFNC were used at baseline according to disease severity.

The HRCT allowed to describe the presence of abnormalities and define a total severity score (TSS) according to Chung, as the sum of the extension of acute lung inflammatory lesions in each of the five lobes, with a range from 0 to 20. The HRCT was performed again to follow up the complications. The follow-up was to continue until discharge or death of all subjects. Patients presenting a non-severe ARDS with pneumomediastinum and/or pneumothorax underwent HFNC therapy, while severe ARDS cases underwent HFNC or invasive mechanical ventilation (IMV) based on SpO2, respiratory rate and respiratory muscle fatigue.

The study was approved by the local ethics committee of University of Campania “Luigi Vanvitelli” and A.O. dei Colli in accordance with the 1976 Declaration of Helsinki and its later amendments. All subjects consented to participate.

Results are reported as number and percentage for categorical variables and median and interquartile range (IQR) for continuous variables. The baseline characteristics reported in Table 1 were compared with a *t*-test. The probability of a severe ARDS with pneumomediastinum in presence of symptoms or not, and subcutaneous emphysema or not was tested by an odds ratio. The recovery rate from the complicating event between HFNC and IMV was tested by a two-tailed Fisher’s exact test. A *p*-value < 0.05 was considered statistically significant.

## 4. Results

Pneumomediastinum/pneumothorax developed in 10.5% of subjects affected by SARS-CoV2-ARDS. Out of 45, 43 patients were male. Median age was 68 years. Of these, 9 subjects had a BMI > 30, 9 subjects showed emphysema at baseline and 1 subject had a previous allergic asthma diagnosis. Common symptoms at COVID-19 onset were fever (82%), dyspnea (40%) and cough (37%). A 59-years old male experienced cough and chest pain, the CT scan showed bilateral pneumothorax at onset of the disease. Moreover, a 66 years-old female showed subcutaneous emphysema and pneumomediastinum at onset. None of the 2 were receiving oxygen or ventilation at home. 

**Figure 1:**
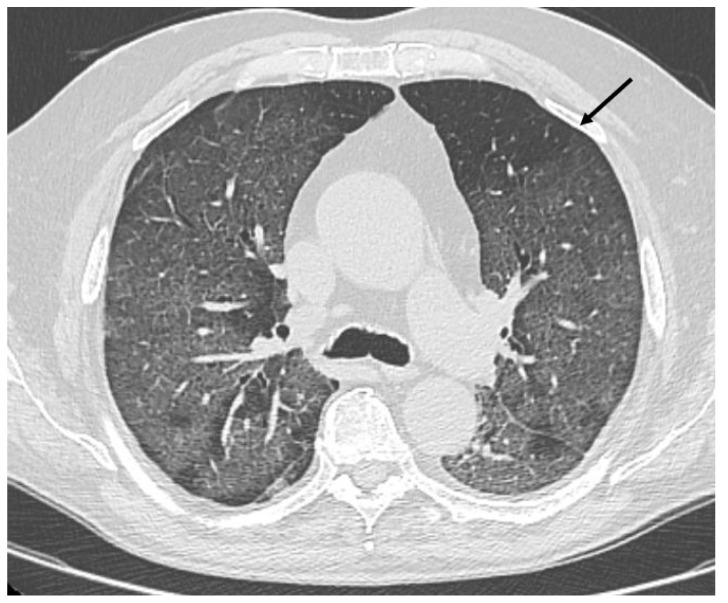
Diffuse bilateral ground glass opacities. Uneven distribution in the upper left lobe (arrow).

**Figure 2:**
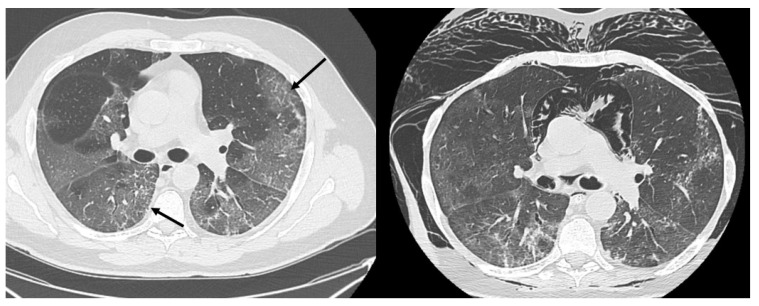
Reticular pattern (arrows) at baseline and massive pneumomediastinum after non-invasive ventilation in a 60-year-old man.

**Figure 3:**
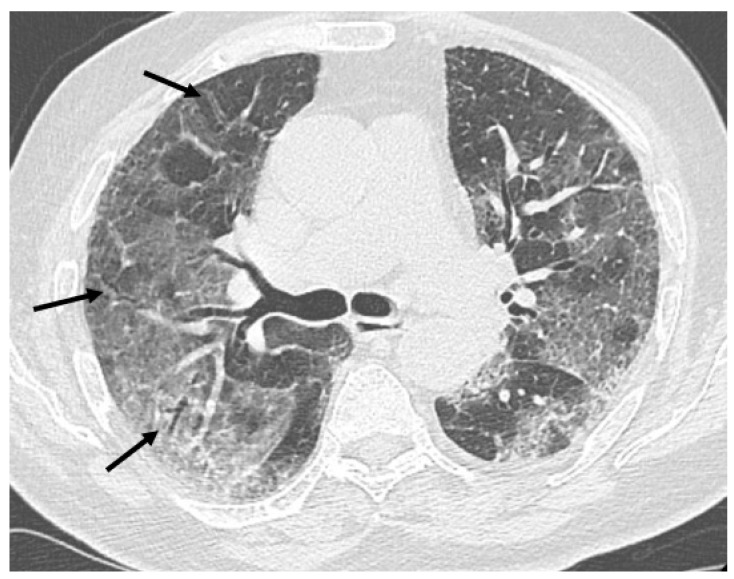
Bronchiolectasis (arrows) in the right lung.

**Figure 4:**
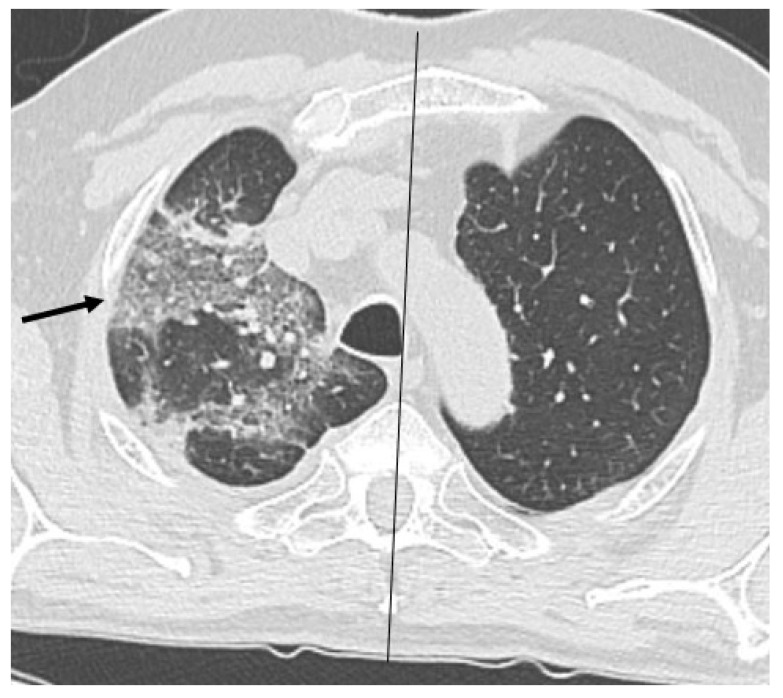
Crazy paving (arrow) and loss of volume of the upper right lobe.

HRCT of the chest showed diffuse bilateral ground glass opacities (GGO) in all patients (Figure 1). Reticulation (Figure 2), traction bronchiectasis/bronchiolectasis (Figure 3), crazy paving and distortion (Figure 4) were observed in 29 patients (64%) at baseline. The total severity score was significantly higher at baseline in patients who eventually developed severe versus non-severe ARDS and pneumomediastinum/pneumothorax (16 versus 14; 95% CI 0.42 to 3.88; *p* = 0.01), as reported in Table 1. No other significant difference was observed at baseline.

At hospital admission all subjects needed ventilatory support. As a first choice, they underwent non-invasive ventilation. As a result, 27 patients received continuous positive airway pressure (CPAP) ventilation via oronasal or total face mask, 11 patients received helmet CPAP and 7 had HFNC at baseline, as reported in Table 2.

The most common complication was pneumomediastinum. It occurred alone in 25 subjects; in 16 cases it was associated with pneumothorax. Pneumothorax alone was observed in 4 cases. In 28 cases (62%), the diagnosis of pneumomediastinum and/or pneumothorax was suspected based on clinical conditions. Moreover, 16 subjects (36%) showed subcutaneous emphysema. Additionally, 3 subjects experienced sinus tachycardia, and 3 had a supraventricular tachyarrhythmia. Chest pain was only referred in 4 cases. Finally, 17 patients were completely asymptomatic. The presence of symptoms is not significantly associated with a severe versus non-severe ARDS and pneumomediastinum/pneumothorax with an odds ratio of 1.83 (95% CI: 0.53 to 6.33; *p* = 0.33). On the other hand, subcutaneous emphysema suggests a higher risk of a severe complication with an odds ratio of 4.89 (95% CI 1.31 to 18.26; *p* = 0.01).

Non-severe cases (*n* = 25) received HFNC set on 34° Celsius and a flow range from 40 to 60 L/min. The fraction of inhaled oxygen (FiO2) ranged between 55% and 100% to maintain a SpO2 ≥ 93%. As a result, 19 patients recovered from pneumomediastinum/pneumothorax over a median follow up of 5 days. While 6 patients (24%) died.

Severe cases received HFNC (*n* = 10) or IMV (*n* = 10). HFNC flow was set between 40–60 L/min and FiO2 ranged between 75–100%. IMV was set on bilevel mode with a FiO2 range between 70–100%. Among HFNC group, 7 patients recovered from the complicating event. Among IMV group, only 1 subject recovered from pneumomediastinum.

## 5. Discussion

The Macklin phenomenon explains how a great pressure gradient between alveoli and interstitium leads to air leak in the thin bronchovascular sheath causing pulmonary interstitial emphysema and ultimately pneumomediastinum. This circumstance can originate from an increase of intrathoracic pressure, as observed during cough or Valsalva maneuver. It reflects on intra-alveolar pressure and overdistension with rupture of alveoli. The resulting air leaks through interstitium towards a lower pressure part of the chest such as mediastinum. Mechanical ventilation is responsible for a higher alveolar end-expiratory pressure which can over-distend alveoli and decrease the venous caliber resulting in a ventilation-perfusion mismatch. 

Low tidal volume lung protective ventilation is a cornerstone of supportive therapy in patients with ARDS, resulting in a significant improvement of the outcomes likely through mitigating the risk of induced lung injury (VILI). In spontaneously breathing patients with lung damage and reduced gas exchange, abnormal respiratory drive with large tidal volumes can exacerbate lung injury in a similar way, termed patient self-inflicted lung injury (P-SILI) [10]. The pathophysiologic basis has been postulated to be the result of a large fluctuation of transpulmonary pressure (P_L_) causing the delivery of quite high tidal volumes and overdistention of the smaller aerated and larger dependent atelectatic lung compartments [11]. Brisk diaphragm contraction may lead to intense local variations in P_L_ predominantly affecting dependent lung, leading to additional stress with the drawing in of gas in from other non-dependent regions (pendelluft phenomenon) and compression with exhalation (atelectrauma) [12]. Additionally, pulmonary capillary vascular pressure and blood flow is augmented from large deflections of P_L_ favoring the formation of pulmonary edema in injured lung units [13]. The harms of dysregulated high respiratory drive thus may lead to a cycle of continued lung injury, respiratory failure and possibly worse outcomes; potentially mitigated by early intubation. 

Although intriguing and plausible, there is currently no prospective trial that offers conclusive evidence to this theory. Precise clinical measures defining P-SILI are also not established, but it still remains an important clinical construct. In an experimental model with spontaneously breathing animals, P-SILI was avoided with the application of a high PEEP; which led to recruitment of dependent lung regions, reduction of big P_L_ changes and decreased inflammation seen on positron emission tomography [14].

Based on that, it is uncertain whether HFNC therapy in patients at risk for P-SILI is helpful or harmful. Nevertheless, we described two cases of pneumomediastinum at onset of disease, in non-ventilated patients, thus suggesting that other mechanisms may predispose COVID-19 patients to certain complicating events. Furthermore, seven cases occurred on HFNC which is an open system delivering quite low positive end-expiratory pressure (PEEP) [15].

The majority of our patients had no smoking history and emphysema affected only nine subjects. Cough was referred by 37% of subjects and can aggravate the risk of potentially negative outcome. Since then, further investigation is required to assess the indication for anti-tussive and sedatives being routinely used in awake patients to control cough. Laxatives should also be tested in order to avoid prolonged Valsalva maneuver. 

From a clinical point of view, ARDS is typically characterized by an increased respiratory flow demand. The rationale for use of HFNC in acute hypoxemic respiratory failure is to reach this demand and reduce work of breathing. This scope is crucial in critical COVID-19, especially when complicating events modify ventilatory mechanics. In fact, in awake patients with ARDS, a ventilatory pattern with large tidal volumes causes major fluctuations of transpulmonary pressure leading to overdistension of alveoli. It has been postulated to exacerbate a patient self-inflicted lung injury (P-SILI). On the contrary HFNC has been shown to create maximal expiratory nasopharyngeal pressures of 5 cm H20 at 50 L/M flow rates, that are unlikely to be sufficient to recruit dependent lung regions [16]. Moreover, the same authors stated that HFNC delivers a variable pressure during the respiratory cycle with a maximum in early expiration. This mechanism supplies a PEEP thus promoting alveolar recruitment but also avoids alveolar overdistension even with a high flow. At the same time, in a small, randomized cross-over study the respiratory mechanics of HFNC at various flow rates were compared to facemask in subjects with hypoxemic respiratory failure. A flow of 60 L/M demonstrated to significantly decrease transesophageal pressure fluctuations indicating patient effort and work of breathing [17]. This would suggest that in some patients HFNC may be adequate therapy to remit the risk of P-SILI.

Clinical measurements of work of breathing or respiratory drive including use of esophageal balloon manometry are cumbersome and impractical in spontaneously breathing patients with lung injury. Without clear guidance or endpoints, a decision to initiate or continue HFNC therapy to prevent lung injury rests on clinical judgment and gestalt. The incorporation of clinical scores predicting failure of HFNC may also be useful to discriminate in this setting.

It has been reported that pneumomediastinum in SARS-CoV2 ARDS may be related to lung frailty more than barotrauma [18]. In our population lung involvement assessed by HRCT revealed an extensive GGO. The median total severity score was 16/20, so that in each lobe coexist pathological and normal groups of alveoli. This condition implies an uneven distribution of pressure during ventilation. In addition, frequently observed abnormalities include reticulation and bronchiolectasis which suggest a parenchymal distortion and possibly a reduction of lung compliance. Crazy paving is an interlobular and intralobular septal thickening, also common in ARDS. It is sometimes a cause of parenchymal distortion eventually leading to loss of lung volume and impaired compliance. We suggest that interstitial pneumonia can lead to architectural distortion and reduced compliance. This reflects on recruitability, in fact, in this condition lungs can tolerate a small variation or pressure, or driving pressure during non-invasive ventilation.

In this study we highlight that 74% of pneumomediastinum resolved after HFNC therapy. This observation suggests that HFNC is a safe ventilatory support for critical COVID-19 and has a potential role in associated complications. Despite this, its use remains controversial and further investigations are needed. The results of this study are limited by the small size cohort and the lack of a randomization.

## 6. Conclusions

HFNC is a safe and effective ventilatory support for critical COVID-19 and has a positive role in associated complications such as pneumomediastinum and pneumothorax. Antitussive and sedatives should be routinely prescribed to cure cough as well as to control abnormal ventilatory mechanics.

## Figures and Tables

**Table 1:** Baseline characteristics.

	Overall Cohort	Mild–Moderate ARDS and Pneumomediastinum/Pneumothorax	Severe ARDS and Pneumomediastinum/Pneumothorax
Total Number	45	25	20
Age Median (IQR)	68 (58–72)	68 (59–74)	63 (57–70)
Emphysema Number	9	8	1
Total severity score Median (IQR)	16 (12–18)	14 (12–16)	16 (15.5–18)
Ground glass opacities Number (Percentage)	45 (100%)	25 (100%)	20 (100%)
Distortion/traction Number (Percentage)	29 (64%)	17 (68%)	12 (60%)
Consolidations Number (Percentage)	11 (24%)	5 (20%)	6 (30%)

**Table 2:** Type of ventilation and symptoms at onset of pneumomediastinum/pneumothorax.

	Overall Cohort (Number)	Mild–Moderate ARDS and Pneumomediastinum/Pneumothorax (Number)	Severe ARDS and Pneumomediastinum/Pneumothorax (Number)
Mask CPAP	27	15	12
Helmet CPAP	11	5	6
HFNC	7	6	1
Duration of NIV before complication (median days and IQR)	8 (5–12)	8.5 (6–13)	7 (5–10)
Symptoms	28	14	14
Subcutaneous emphysema	16	5	11
No symptoms	17	11	6
